# Surgical outcomes of ureteral reconstruction during cytoreductive surgery for ovarian cancer: a retrospective cohort study

**DOI:** 10.1186/s12885-022-10288-x

**Published:** 2022-11-11

**Authors:** Ji Hyun Kim, Dong-eun Lee, Hyeong In Ha, Jae Young Jung, Sung Han Kim, Hyung Ho Lee, Ho Kyung Seo, Sang-Soo Seo, Sokbom Kang, Sang-Yoon Park, Myong Cheol Lim

**Affiliations:** 1 Center for Gynecologic Cancer, National Cancer Center, 323, Ilsan-Ro, Ilsandong-Gu, Gyeonggi-Do, Goyang-Si, 10408 Republic of Korea; 2 Biostatistics Collaboration Team, National Cancer Center, Goyang, Republic of Korea; 3 Department of Obstetrics and Gynecology, Pusan National University Yangsan Hospital, Pusan National University School of Medicine, Yangsan, Republic of Korea; 4 Department of Urology, National Cancer Center, Goyang, Republic of Korea; 5 Center for Clinical Trials, National Cancer Center, Goyang, Republic of Korea; 6 Department of Tumor Immunology, Research Institute and Hospital, National Cancer Center, Goyang, Republic of Korea; 7 Rare and Pediatric Cancer Branch and Immuno-Oncology Branch, Division of Rare and Refractory Cancer, Research Institute, National Cancer Center, Goyang, Republic of Korea

**Keywords:** Ovarian cancer, Cytoreductive surgery, Ureteral reconstruction

## Abstract

**Background:**

Ureteral reconstruction is required after surgical resection of the tumor invading the urinary tract in ovarian cancer with low incidence. There are no currently reported surgical outcomes of ureteral reconstruction during cytoreductive surgery. The aim of the study is to investigate the clinical features and surgical outcomes of ureteral reconstruction during primary, interval and secondary cytoreductive surgery for ovarian cancer.

**Methods:**

A total of 3226 patients who underwent primary, interval or secondary cytoreductive surgery for ovarian cancer between January 2000 and May 2021 were reviewed. Fifty-six patients who underwent ureteral reconstruction during cytoreductive surgery were included in the analysis.

**Results:**

Ureteral reconstruction was required in 1.7% (56/3226) of ovarian cancer patients. Of the 56 patients who underwent ureteral reconstruction during cytoreductive surgery, 35 (62.5%) had primary ovarian cancer, and 21 (37.5%) had recurrent ovarian cancer. The median tumor size invading the lower urinary tract was 2.0 cm (range, 0.4–9.5 cm). Ureteroneocystostomy with direct implantation (51.8%) and psoas hitch (8.9%), transureteroureterostomy (7.1%), and ureteroureterostomy (32.1%) were required as part of cytoreductive surgery. Complete cytoreduction with ureteral reconstruction was achieved in 83.9% (47/56) and the rest of the patient population (16.1%) achieved a gross residual tumor size of less than 1 cm. All complications, including hydronephrosis (33.9%), were managed, none resulting in long-term sequelae. In primary ovarian cancer, the 5-year disease-free survival and overall survival were 50.0% and 89.5%, respectively. In patients with recurrent ovarian cancer, the 5-year disease-free survival and overall survival were 23.6% and 64.0%, respectively.

**Conclusions:**

Ureteral reconstruction as a part of cytoreductive surgery for ovarian cancer could be performed with acceptable morbidities. Complete cytoreduction by a multidisciplinary surgical team, including urologic oncologists, should be pursued for the surgical management of ovarian cancer.

**Trial registration:**

Retrospectively registered.

**Supplementary Information:**

The online version contains supplementary material available at 10.1186/s12885-022-10288-x.

## Introduction

Ovarian cancer is one of the most lethal gynecologic malignancies, and approximately 75% of all newly diagnosed patients are diagnosed in the advanced stage [1, 2]. According to worldwide cancer statistics, the incidence and mortality of ovarian cancer were estimated to be 313,959 and 207,252, respectively, in 2020 [1].

Although there has been significant progress in the treatment of ovarian cancer over recent decades, complete cytoreductive surgery remains one of the most important factors for improving survival outcomes in primary advanced ovarian cancer [3, 4]. Additionally, we recently reported that secondary cytoreductive surgery with complete gross resection resulted in survival benefits in platinum-sensitive recurrent ovarian cancer from a meta-analysis that included three randomized trials [5].

Ureteral reconstruction is required after surgical resection of the tumor invading the urinary tract in ovarian cancer with low incidence [6–8]. If the ovarian tumor grows massively as in cases of advanced disease, there is a high risk of ureteric invasion or damage when attempting *en bloc* resection of the tumor because of the proximity between the ovaries and urological organs [9]. Even with early-stage ovarian cancer, urinary tract surgery might be required due to several factors, including adhesion or anatomical variations. For instance, deep ureteral infiltration of endometriosis is one contributing factor toward considering ureteral reconstruction [10].

Existing data concerning the surgical outcomes of ureteral reconstruction during cytoreductive surgery for ovarian cancer and its complications are limited by small sample-size cases. *Berek et al.* reported that 24 (2.8%) of 848 patients underwent lower urinary tract resection during primary or secondary cytoreduction [7]. The median overall survival (OS) was 12 months, and 16 (66.7%) patients had residual tumors less than 2 cm in size. Further, six major complications, including urinary stricture and kidney atrophy, which necessitate re-anastomosis, have also been reported [7]. Additionally, *Malviya et al.* reported survival outcomes of twenty-two patients who underwent urinary tract resection. The mean OS was 15.2 months, and nine major complications, including early postoperative death, were observed [11]. There is currently no report related to surgical management of urinary tract as part of cytoreduction for ovarian cancer.

The study aimed to evaluate the postoperative outcomes of ureteral reconstruction during cytoreductive surgery for ovarian cancer.

## Materials and methods

Between January 2000 and May 2021, patients who underwent ureteral reconstruction, including ureteroneocystostomy, end to end ureteroureterostomy, and transureteroureterostomy during cytoreductive surgery were eligible for inclusion. Patients’ data, including epidemiologic characteristics, tumor stage at diagnosis, surgical records, residual tumor after cytoreductive surgery, and records of adjuvant chemotherapy, were extracted from electronic medical records, and retrospectively reviewed. Of 3226 patients who underwent primary, interval or secondary cytoreductive surgery, 56 patients were included in the study. The study was approved by institutional review board of our organization.

Ureteral reconstruction was performed by four urologists. There could be additional procedures, including psoas hitch or Boari flap, and after anastomosis of urinary tract is completed, a ureteral stent was inserted to secure patency of the anastomosis.

The extent of tumor metastasis, accompanying procedures, and type of ureteral reconstruction were assessed from the medical records. Additionally, operating time with perioperative blood loss, duration of hospital stays, antibiotic use, and time interval from surgery to initiation of chemotherapy were estimated. After ureteral reconstruction, insertion of a ureteral stent is usually mandatory, when applicable. The interval from surgery to removal of the ureteral stent was also assessed. Postoperative adverse events were assessed until 30 days after cytoreduction, and grade classification followed the Common Terminology Criteria for Adverse Events version 5.0.

Patients’ characteristics and surgical features were presented as categorical variables using frequencies and proportions, and continuous variables were presented as medians and ranges. disease-free survival (DFS) was measured as the interval from the date of cytoreductive surgery to the date of cancer recurrence, death, or last contact. OS was defined as the duration from cytoreductive surgery until death. Kaplan–Meier curves were generated for DFS and OS.

Statistical significance was assumed for *P* < 0.05. All statistical analyses were conducted using SAS software, version 9.4 (SAS Institute Inc, Cary, NC, USA.) and R software, version 4.1.2 (R Foundation for Statistical Computing, Vienna, Austria.).

## Results

Ureteral reconstruction was required in 1.7% (56/3226) of ovarian cancer patients. Of the 56 patients who underwent ureteral reconstruction during cytoreductive surgery, 35 (62.5%) had primary ovarian cancer and 21 (37.5%) had recurrent ovarian cancer. In patients with primary ovarian cancer, eight (14.3%) patients underwent interval cytoreductive surgery. The baseline characteristics of the patients are described in Table 1. The median age at diagnosis was 54 years (range, 18–73 years), and most patients had Eastern Cooperative Oncology Group performance status 0–1 (96.4%). The distribution of surgical stage was as follows: 7 (12.5%) for stage I, 7 (12.5%) for stage II, 33 (58.9%) for stage III, and 9 (16.1%) for stage IV.

**Table 1:** Patient characteristics for primary or recurrent ovarian cancer who underwent ureteral reconstruction

Characteristics	Total	Primary	Recurrent
	*N* = 56 (%)	*N* = 35 (62.5%)	*N* = 21 (37.5%)
**Median Age at diagnosis, years** (range)	54 (18–73)	52 (39–73)	51 (18–73)
**BMI, kg/m2** (range)	22.3 (16.2–31.7)	20.1 (16.4–31.7)	21.8 (16.2–29.7)
**ECOG performance status**			
0–1	54 (96.4)	34 (97.1)	20 (95.2)
2	2 (3.6)	1 (2.9)	1 (4.8)
**Stage (FIGO 2014)**^a^			
I	7 (12.5)	6 (17.1)	1 (4.8)
II	7 (12.5)	4 (11.4)	3 (14.3)
III	33 (58.9)	20 (57.1)	13 (61.9)
IV	9 (16.1)	5 (14.3)	4 (19.0)
**Histology**			
High grade serous	27 (48.2)	18 (51.4)	9 (42.9)
Endometrioid	7 (12.5)	6 (17.1)	2 (9.5)
Clear cell	7 (12.5)	4 (11.4)	3 (14.3)
Mucinous	2 (3.6)	1 (2.9)	2 (9.5)
Neuroendocrine carcinoma	1 (1.8)	1 (2.9)	0 (0)
Granulosa cell tumor	3 (5.4)	1 (2.9)	2 (9.5)
Carcinosarcoma	3 (5.4)	1 (2.9)	2 (9.5)
Others	5 (8.9)	3 (8.6)	2 (9.5)
**Neoadjuvant chemotherapy**			
No	48 (85.7)	27 (77.1)	21 (100)
Yes	8 (14.3)	8 (22.9)	0 (0)
**Previous radiotherapy**			
No	55 (98.2)	35 (100)	20 (95.2)
Yes	1 (1.8)	0 (0)	1 (4.8)
**Previous chemotherapy**			
No	34 (60.7)	32 (91.4)	2 (9.5)
Yes	22 (39.3)	3 (8.6)	19 (90.5)

^a^Initial FIGO stage was considered at the time of primary ovarian cancer

High-grade serous ovarian cancer (48.2%) was the most common histology, followed by endometrioid (12.5%) and clear cell carcinoma (12.5%). Nineteen (90.5%) patients with recurrent ovarian cancer had previous chemotherapy, and one with granulosa cell tumor underwent radiotherapy but later experienced recurrence (Table 1).

Surgical indication for ureteral surgery was as follows: Ureterovesical invasion by the tumor in 52 (92.9%) patients. Intraoperative injury was the additional indication for ureteral reconstruction in four (7.1%) patients (Table 2). The median tumor size invading the lower urinary tract was 2.0 cm (range, 0.4–9.5 cm). The size of the tumor was more than 1 cm in diameter in 43 (76.8%) patients. Complete cytoreductive surgery with microscopic residual tumor was achieved in 47 (83.9%) patients and the rest of the patient population (16.1%) achieved a gross residual tumor size of less than 1 cm. The residual tumors were located at the small bowel mesentery and were 1–3 mm in diameter.

**Table 2:** Perioperative and pathological features in primary or recurrent ovarian cancer patients with ureteral reconstruction

Variables	Total	Primary	Recurrent
	*N* = 56 (%)	*N* = 35 (62.5%)	*N* = 21 (37.5%)
**Indications for Ureteral surgery**			
Ureterovesical invasion by tumor	52 (92.9)	32 (91.4)	20 (95.2)
Intraoperative injury	4 (7.1)	3 (8.6)	1 (4.8)
**Size of tumor at the ureterovesical area**			
< 1 cm	13 (23.2)	7 (20.0)	6 (28.6)
≥ 1 cm	43 (76.8)	28 (80.0)	15 (71.4)
**Residual tumor**			
Microscopic	47 (83.9)	29 (82.9)	18 (85.7)
< 1 cm	9 (16.1)	6 (17.1)	3 (14.3)
≥ 1 cm	0 (0)	0 (0)	0 (0)
**Presence of endometriosis**			
No	45 (80.4)	26 (74.3)	8 (38.1)
Yes	11 (19.6)	9 (25.8)	2 (9.5)
**Accompanied surgical procedures**			
Hysterectomy	30 (53.6)	29 (82.9)	1 (4.8)
Bilateral salpingo-oophorectomy	32 (57.1)	30 (85.7)	2 (9.5)
Pelvic Lymphadenectomy	40 (71.4)	33 (94.3)	7 (33.3)
Para-aortic Lymphadenectomy	40 (71.4)	33 (94.3)	7 (33.3)
Omentectomy	40 (71.4)	29 (82.9)	11 (52.4)
Splenectomy	10 (28.6)	5 (14.3)	5 (23.8)
Rectosigmoid resection	40 (71.4)	28 (80.0)	12 (57.1)
Ileostomy	5 (8.9)	4 (11.4)	1 (4.8)
Small bowel resection	13 (23.2)	6 (17.1)	7 (33.3)
HIPEC	5 (8.9)	5 (14.3)	0 (0)
**Types of Ureteral surgery**			
Ureteroneocystostomy—Direct reimplantation	29 (51.8)	19 (54.3)	10 (47.6)
Ureteroneocystostomy—Psoas hitch	5 (8.9)	2 (5.7)	3 (14.3)
Transureteroureterostomy	4 (7.1)	2 (5.7)	2 (9.5)
Ureteroureterostomy	18 (32.1)	12 (34.3)	6 (28.6)
**GFR outcome, mL/min/1.73m**^**2**^**; mean**			
Pre-Op GFR (MDRD)	77.9	80.7	73.4
Post-Op GFR (MDRD)	89.1	88.9	89.5
% Changes	14.3	10.2	21.9
**Operation time, hours**; median (min–max)	7 (3–12)	6 (3–12)	5 (4–10)
**Blood loss, mL**; median (min–max)	800 (200–5700)	500 (300–5700)	500 (200–1600)
**Interval from operation to adjuvant chemotherapy (days)**; median (min–max) (missing = 4)	24 (10–62)	24 (11–40)	27 (10–62)
**Day of stent removal**; median (min–max)	40 (13–322)	43 (13–241)	29 (21–322)
**Hospital stays, days**; median (min–max)	22 (5–63)	24 (9–63)	19 (10–63)
**Perioperative RBC transfusion, unit**; median (min–max)	1 (0–9)	2 (0–9)	0.5 (0–5)
**Antibiotics use, days;** median (min–max)	6 (2–59)	10 (2–59)	3 (2–25)

*HIPEC* Hyperthermic intraperitoneal chemotherapy, *GFR* glomerular filtration rate, *MDRD* Modification of Diet in Renal Disease Study, *RBC* Red blood cells

**Fig. 1:**
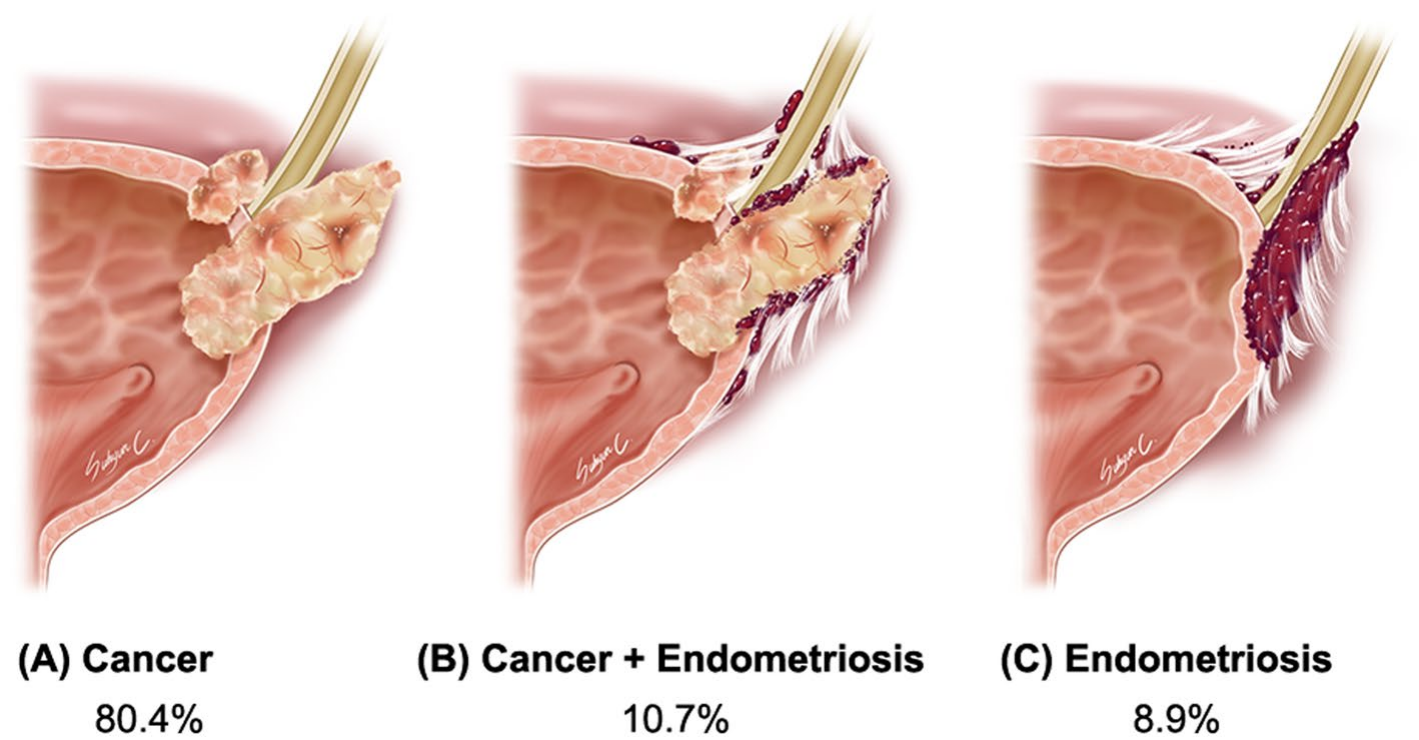
Proportion of the presence of endometriosis. The pathologic outcome of ureterovesical tumor was as follows: cancer without endometriosis in 45 (80.4%) patients, combined involvement of endometriosis and cancer in 6 (10.7%), and endometriosis without cancer in 5 (8.9%)

When the pathologic outcome of the resected ureterovesical tumor was reported, endometriosis was found in eleven (19.6%) patients. Of the eleven patients, endometriosis without cancer was found in five (8.9%), and the combined involvement of endometriosis and cancer was found in six patients (10.7%) (Fig. 1).

Procedures accompanied with ureteral surgery are listed in Table 2. The majority of patients with primary ovarian cancer underwent a hysterectomy (82.9%), bilateral salpingo-oophorectomy (85.7%), omentectomy (82.9%), and pelvic (94.3%) and para-aortic lymphadenectomy (94.3%). Furthermore, five (14.3%) patients underwent splenectomy, and 28 (80.0%) underwent rectosigmoid resection concomitantly with ureteral reconstruction for primary ovarian cancer. Regarding recurrent ovarian cancer, twelve (57.1%) patients underwent rectosigmoid resection, with small bowel resection in seven (33.3%).

**Fig. 2:**
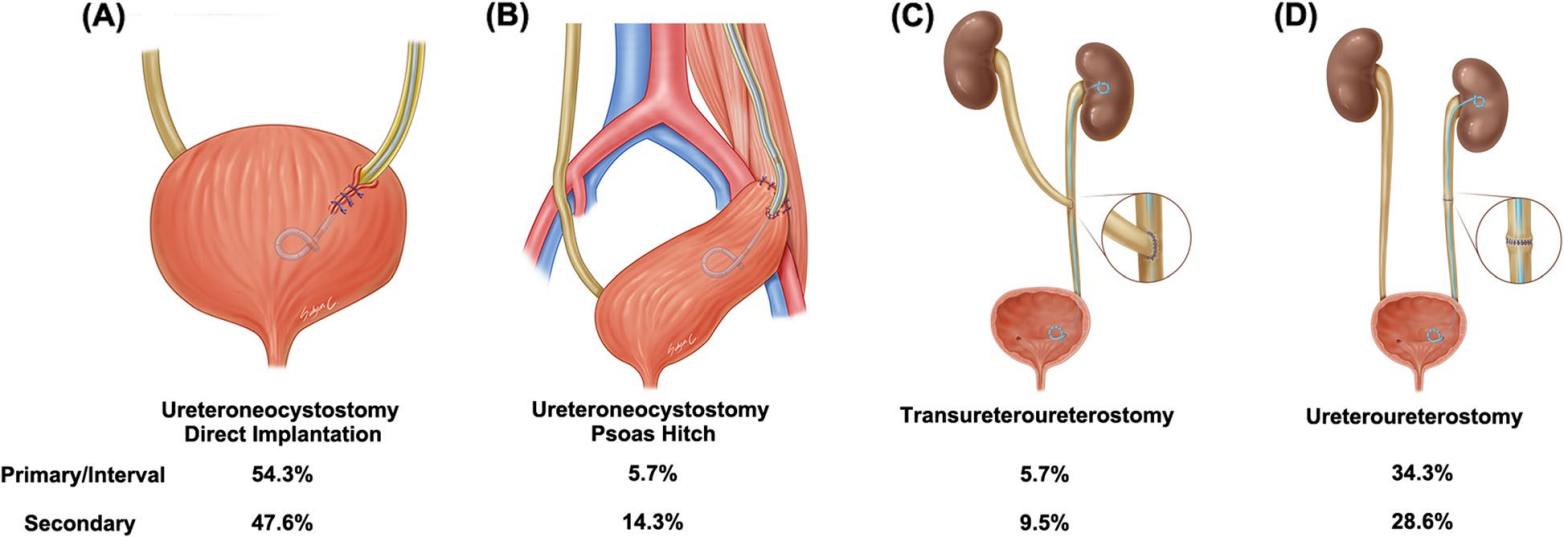
Surgical type of ureteral reconstruction (**a**) Ureteroneocystostomy with direct reimplantation (**b**) Ureteroneocystostomy with psoas hitch procedure: indicated when the distal ureter was resected and the remaining portion of ureter did not reach the bladder. The bladder was pulled up and fixed to the psoas muscle by two or three hitches of suture to achieve a tension-free re-implantation of the ureter. (**c**) Transureteroureterostomy: Resected ureter is joined to another ureter (**d**) Ureteroureterostomy

Four types of ureteral reconstruction were performed during primary or secondary cytoreductive surgery; direct reimplantation of ureteroneocystostomy (51.8%), psoas hitch procedure with ureteroneocystostomy (8.9%), transureteroureterostomy (7.1%) and ureteroureterostomy (32.1%) (Fig. 2). Psoas hitch technique was additionally performed in patients when the length of the resected ureter was ranged from 4.0 cm to 6.3 cm.

The mean pre- and postoperative glomerular filtration rate using Modification of Diet in Renal Disease (MDRD) formula were 77.9 ml/min/1.73m^2^ and 89.1 ml/min/1.73m^2^, respectively. Renal function was not worsened after ureteral reconstruction in entire cohort. The median operative time was 7 h, and the median estimated blood loss was 800 ml. In addition, the median interval time from cytoreductive surgery to adjuvant chemotherapy was 24 days, and median interval from surgery to stent removal was 40 days.

The postoperative adverse events are summarized in Table 3. In our study, twenty-eight patients developed at least one postoperative complication. Grade 2 ureteral anastomotic leak occurred in two (3.6%) patient, grade 2 ureteral stricture in two (3.6%), and grade 3 fistula in two (3.6%). Anastomotic leakage and urinary fistula were treated with percutaneous nephrostomy and intravenous antibiotic use, and ureteral stricture was managed by retaining the ureteral stent for 35–60 days.

**Table 3:** Postoperative adverse events in primary or recurrent ovarian cancer patients with ureteral reconstruction

Adverse event	Total (*N* = 56)	Primary (*N* = 35)		Recurrent (*N* = 21)	
	Grade 1–4	Grade 1–2	Grade 3–4	Grade 1–2	Grade 3–4
**Ureterorenal**					
Ureteral anastomotic leak	3 (5.4)	1 (1.8)	0 (0)	1 (1.8)	1 (1.8)
Ureteral stricture	2 (3.6)	1 (1.8)	0 (0)	1 (1.8)	0 (0)
Urinary fistula	3 (5.4)	2 (3.6)	1 (1.8)	0 (0)	0 (0)
Hydronephrosis	19 (33.9)	8 (14.3)	7 (12.5)	3 (5.4)	1 (1.8)
Acute renal failure	4 (7.1)	0 (0)	1 (1.8)	2 (3.6)	1 (1.8)
**Infection**					
Urinary tract infection	20 (35.7)	12 (21.4)	3 (5.4)	1 (1.8)	4 (7.1)
Wound infection	9 (16.1)	4 (7.1)	2 (3.6)	2 (3.6)	1 (1.8)
Sepsis	2 (3.6)	0 (0)	1 (1.8)	0 (0)	1 (1.8)
**Respiratory**					
Pleural effusion	8 (14.3)	5 (8.9)	0 (0)	3 (5.4)	0 (0)
Pneumonia	0 (0.0)	0 (0.0)	0 (0.0)	0 (0.0)	0 (0.0)
**Cardiovascular**					
Thromboembolic event	2 (3.6)	2 (3.6)	0 (0)	0 (0)	0 (0)
Lymphocele	17 (30.4)	9 (16.0)	3 (5.4)	3 (5.4)	2 (3.6)

Postoperative hydronephrosis was observed in 19 (33.9%) patients; however, after three cycles of chemotherapy, all cases of hydronephrosis resolved. Grade 2 and 3 acute renal failure occurred in two (3.6%) patients, respectively. In addition, urinary tract infection was observed in twenty (35.7%) patients, thirteen of which were grade 2, and six were grade 3, while grade 4 sepsis occurred in one (1.8%) patient. The patient who had urosepsis simultaneously developed anastomotic leakage and was treated with antibiotic use and percutaneous nephrostomy. Lymphocele was found in 17 (30.4%) patients, and grade 3 lymphocele in five (8.9%) patients was treated with percutaneous drainage insertion and antibiotic use (Table 3).

**Fig. 3:**
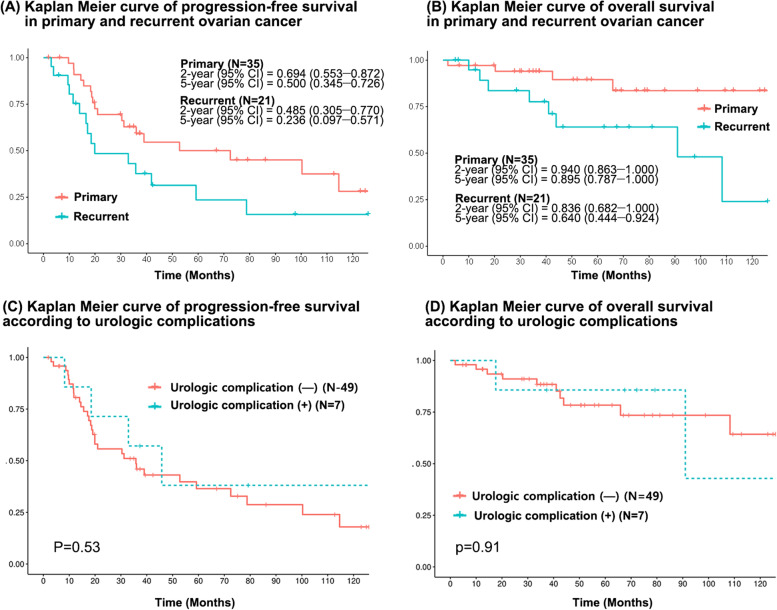
Kaplan–Meier curve for progression-free survival (DFS) and overall survival (OS) (**a**) Kaplan–Meier curve for DFS in primary and recurrent ovarian cancer (**b**) Kaplan–Meier curve for OS in primary and recurrent ovarian cancer (**c**) Kaplan–Meier estimates for DFS according to urologic complications (**d**) Kaplan–Meier estimates for OS according to urologic complications

The median duration of follow-up for all patients was 46.6 months. Regarding patients with primary ovarian cancer, the 2-year DFS was 69.4%, and the 5-year DFS was estimated to be 50.0% (Fig. 3a). In patients with recurrent ovarian cancer, the 2-year DFS was 48.5%, and the 5-year DFS was estimated to be 23.6% (Fig. 3a). In patients with primary ovarian cancer, the 2-year OS was 94.0% and the 5-year OS was 89.5% (Fig. 3b), while in patients with recurrent ovarian cancer, the 2-year OS was 83.6 and the 5-year OS was 64.0% (Fig. 3b). There was no statistically significant difference in DFS (*p* = 0.53) and OS (0.91) between the patients with or without postoperative urologic complications, including ureteric fistula, stricture, or acute renal failure (Fig. 3c, 3d).

## Discussion

In the current study, 56 patients underwent ureteral reconstruction as part of cytoreductive surgery with acceptable and manageable complications. Compared with two previous studies presenting surgical outcomes of ureteral surgery in ovarian cancer, major complications were significantly minimized and successfully managed [7, 11]. There was no perioperative mortality in this study, and there was one grade 4 adverse event of sepsis.

Several studies have analyzed the surgical outcomes of ureteral surgery in other cohorts, including colorectal cancer, as described in Table S1. In a colorectal cancer study by *Heijkant *et al., urinary leakage after ureteral reconstruction was identified in 16 (22.9%) patients compared with one (3.4%) in our study [12]. As mentioned in the same study, higher rates of urinary leakage in colorectal cancer might be associated with previous radiotherapy [12, 13]. Forty (57.1%) patients in the colorectal cancer study underwent preoperative radiotherapy, compared with three (5.4%) in our study. In the other cohort with gynecologic malignancy, urinary leakage was identified at a similar rate of 8.6% [14].

In our study, we presented the indications of ureteral reconstruction that were not mentioned in the two previous studies on urinary tract surgery in ovarian cancer. Ureteral reconstruction was primarily required because of ureterovesical tumor invasion (92.9%). In addition, another indication of ureteral reconstruction in four (7.1%) patients was the intraoperative injuries during cytoreductive surgery. Two of patients had endometriosis with severe adhesion in the pelvic cavity, other patients had pelvic adhesion related to prior abdominal surgeries or inflammation.

In this study, the proportion of high-grade serous ovarian cancer and advanced stage ovarian cancer was not as high as expected in women who required ureteral reconstruction. Fourteen (25.0%) patients had a histologic type of either endometrioid or clear cell carcinoma, and these subtypes of ovarian cancer are well-known endometriosis-associated ovarian cancer [15]. The infiltrative characteristic of endometriosis increases the risk of ureteral reconstruction during cytoreductive surgery [16]. In addition, endometriosis was pathologically identified in eleven (19.6%) patients, five of whom had only endometriosis lesion in the invaded tumor, and their stage was I. A previous study demonstrated that endometriosis was frequently identified in the early cancer stage compared with the late stage in clear cell carcinoma [17]. Therefore, surgical management of urinary tract was required in the case of endometriosis-related ovarian cancer.

Ureteral reconstruction with direct anastomosis could be successfully accomplished if the ureteral length is sufficient to create a tension-free anastomosis [18]. If the remnant ureteral length is insufficient to anastomosis without tension, the psoas hitch technique or transureteroureterostomy is needed for a tension-free anastomosis [19]. In our study, most patients (83.9%) favorably underwent direct reanastomosis without additional flap or transureteroureterostomy.

The use of ureteral stents could reduce urologic complications, including postoperative stenosis and leakage at the anastomotic site, as demonstrated by several case–control studies and randomized controlled trials [20–22]. However, prolonged stent retention might predispose to urinary tract infections, encrustations and blockade of stents [23, 24]. In our study, the median interval from ureteral surgery to stent removal was 40 days. The ureteral stent is generally removed after 28 to 42 days after ureteral surgery, [25] and long-term indwelling stents more than 6 weeks was associated with higher ureteral complications [24]. Therefore, proper surveillance for the stent management is needed, and timing of stent removal should be counseled with urologists, considering complications and ureteral resticture risk.

The time interval between cytoreductive surgery and initiation of adjuvant chemotherapy is associated with survival outcomes [26, 27]. In an ancillary study of the Gynecologic Oncologic Group randomized controlled trial by *Tewari *et al., delaying the initiation of chemotherapy for more than 25 days adversely affected OS in patients with advanced ovarian cancer [27]. In the current study, the median interval from ureteral reconstruction to adjuvant chemotherapy was 24 days. Postoperative adverse events related to ureteral surgery, such as anastomotic leakage or urinary fistula, might be considered to negatively affect the recovery time, resulting in the delay of chemotherapy. However, in this study, the addition of ureteral reconstruction as part of cytoreductive surgery did not significantly delay the initiation of adjuvant chemotherapy.

Our study has several limitations. First, the data were retrospectively analyzed; therefore, selection bias or recall bias was inevitable. Second, treatment outcomes, including survival outcomes, from the small number of the participants should be confirmed in larger prospective cohorts. Third, four urologists participated in this surgical series with different levels of surgical experience. However, this study included the largest number of patients with ovarian cancer who underwent ureteral reconstruction, suggesting surgical feasibility with acceptable morbidities.

## Conclusions

In conclusion, our study supports the feasibility of ureteral reconstruction as part of cytoreductive surgery for complete resection of ovarian tumors with no visible residual tumor, with multidisciplinary team. Ureteral reconstruction with urinary tract resection can be safely utilized with acceptable survival outcomes.

## Supplementary Information

**Additional file 1:** **Table S1. **Review of surgical outcomes of urinary tract resection.

## Data Availability

The datasets used in the current study are available from the corresponding author on request.

## Abbreviations

DFS: Disease-free survival
OS: Overall survival
