# Supplementary material for: Surgical outcomes of ureteral reconstruction during cytoreductive surgery for ovarian cancer: a retrospective cohort study
Source: BMC Cancer. 2022 Nov 11;22:1163. doi: 10.1186/s12885-022-10288-x (PMC9650832; doi:10.1186/s12885-022-10288-x)
Supplement: Supplementary file 1 — Additional file 1: Table S1. Review of surgical outcomes of urinary tract resection. [file 12885_2022_10288_MOESM1_ESM.docx]

| **Table S.1.** **Review of surgical outcomes of Urinary Tract Resection** | | | | |  |  |  |  |
| --- | --- | --- | --- | --- | --- | --- | --- | --- |
| Number | Authors, Year | Enrolled patients (primary/recurrent) | Origin of cancer | Study Design | Preoperative radiotherapy or chemotherapy | Residual tumor | Survival | Postoperative Complications |
| 1 | Heijkant et al, 2017 | 70 (30/40) | Rectal cancer | Retrospective study  single arm | Radiotherapy:40 recurrent patients. | N/A | Median OS: 40 months (95%CI, 12.9-73.1) without complications  50 months (95% CI,0.1-102.0) with complications | 38.6% Urinary leakage (22.9%) Urinary stricture (8.6%)  Urosepsis (4.3%) |
| 2 | Federico et al, 2020 | 46 (26/20) | Cervical cancer (N=26) Endometrial cancer (N=4) Ovarian cancer (N=17) Vaginal cancer (N=1) Uterine sarcoma (N=3) | Retrospective study  single arm single institution | Radiotherapy: 12 (26.0%)  Chemotherapy: 8 (17.3%) | R0 - 40 (86.9%) R1 - 6 (13.1%) | Median PFS:  24 months (95%CI, 14.9-33.0) | 26.0% (Early major complication) Urinary leakage  (8.6%) sepsis (6.5%)  Uretero-vaginal fistula (4.3%) |
| 3 | Hackethal et al, 2013 | 21 | Ovarian cancer (N=13) Endometriosis (N=4) Leiomyosarcoma (N=1) Endometrial Cancer (N=1) Cervical Cancer (N=1)  Borderline Ovarian tumor (N=1) | Retrospective study  single arm  single institution | N/A | N/A | N/A | Urinary tract infection (28.6%) Moderate/severe hydronephrosis (21.1%) |
| 4 | Hoffman et al, 2006 | 46 | Cervical cancer (N=2) Ureteral endometriosis (N=1) Ovarian cancer (N=9) Vaginal cancer (N=2) Others (N=2) Not assessed, ureteral injury (N=30) | Retrospective study  single arm single institution | None (0%) | N/A | N/A | Vesicovaginal fistula (2.1%)  Urinary incontinence (2.1%) |
| 5 | Manolistas et al, 2001 | 8 | Ovarian cancer (N=6) Vaginal cancer (N=1) Uterine sarcoma (N=1) | Retrospective study  single arm multicenter institution | N/A | N/A | N/A | Postoperative mortality (1/8, 12.5%) Ureteric obstruction due to abscess (1/8. 12.5%) Urinary tract infection (3/8, 37.5%) |
